# Enhancing the physiological characteristics of chimeric antigen receptor natural killer cells by synthetic biology

**DOI:** 10.3389/fimmu.2025.1592121

**Published:** 2025-04-17

**Authors:** Shuochao Ji, Cheng Jin, Xinjiang Cui

**Affiliations:** ^1^ Affiliated Hospital of Shandong Second Medical University, Weifang, China; ^2^ Department of Hematology and Oncology, Shenzhen Children’s Hospital, Shenzhen, China

**Keywords:** NK cells, chimeric antigen receptor, synthetic biology, immunotherapy, TME

## Abstract

Chimeric antigen receptor natural Killer (CAR-NK) cells therapy represents a next-generation immunotherapeutic approach following CAR-T cells therapy, offering inherent “off-the-shelf” compatibility and mitigated off-tumor toxicity. Despite these advantages, clinical translation remains constrained by poor *in vivo* persistence and functional exhaustion in immunosuppressive tumor microenvironments (TME). This review examines recent advancements in synthetic biology aimed at enhancing the physiological characteristics of CAR-NK cells. By delineating the synergy between NK cells and synthetic biology toolkits, this work provides a roadmap for developing next-generation CAR-NK therapies capable of addressing solid tumor challenges while maintaining favorable safety profiles.

## Introduction

Natural killer (NK) cells constitute critical effectors of innate immunity, deploying multifaceted cytotoxic mechanisms through dynamic integration of activating (e.g., NKG2D) and inhibitory (e.g., KIR, NKG2A) receptors. NK cells execute tumoricidal activity through three mechanistically distinct pathways: perforin/granzyme-mediated lysis; death receptor (FasL/TRAIL)-induced apoptosis; and antibody-dependent cellular cytotoxicity (ADCC) via FcγRIIIa (CD16) engagement (1). CAR-NK cells leverage the innate cytotoxic capacity of NK cells and gain specificity against particular tumor-associated antigens, thus improving their effectiveness in targeting malignant cells. Distinct from CAR-T cells, CAR-NK cells maintain dual targeting mechanisms both through their engineered receptors and their native activating receptors. Notably, CAR-NK cells do not require human leukocyte antigen (HLA) matching (2), maximumly eliminate graft-versus-host disease and cytokine release syndrome risks (3), and showed superior TME infiltration capabilities (4). Moreover, there are broader sources of NK cells (5), making them suitable for “off-the-shelf” therapeutic applications. This feature significantly enhances their accessibility and reduces the time required for treatment preparation, which is crucial for patients with aggressive cancers. Despite these merits, CAR-NK cells face cardinal translational barriers in terms of transient persistence and TME-imposed suppression (6). To address these challenges, researchers are exploring various approaches that can enhance the durability and effectiveness of CAR-NK cells. Synthetic biology approaches showed promising effects in CAR-NK cells therapy by focusing on the design, construction, and assembly of modular components. Validating these innovations in clinical trials will bridge current limitations to therapeutic application.

## NK cells biology and sources

**Figure 1:**
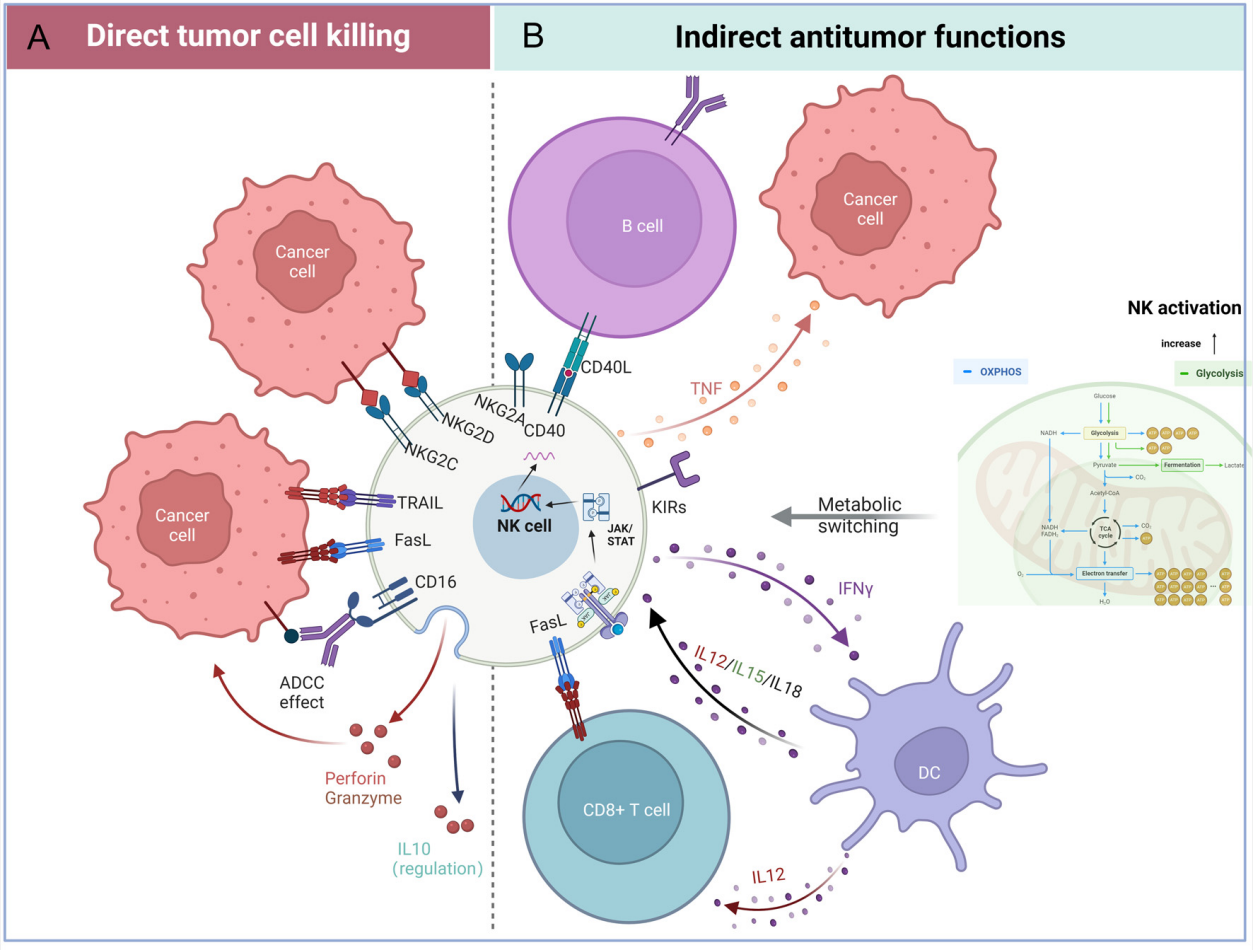
Functional attributes of natural killer (NK) cells in tumor immunity. **(A)** Direct tumor-killing mechanisms: NK cells eliminate cancer cells through activating receptors (e.g., NKG2D), ADCC via CD16, and the FasL/TRAIL-mediated death pathways. **(B)** Indirect antitumor effects: NK cells secrete immunomodulatory factors, including interferon-gamma (IFN-γ), cytokines, and chemokines, to recruit and activate adaptive immune cells. Additionally, metabolic reprogramming in the tumor microenvironment enhances NK cell effector functions. (Figure created with BioRender).

NK cells constitute critical effectors of the innate immune system, characterized by their unique capacity to detect and eliminate malignant or infected cells through a sophisticated balance of activating and inhibitory receptors (7). Notably, NK cells also mediate ADCC via CD16 engagement with antibody-opsonized targets, triggering cytotoxic granule polarization and release (8) (**Figure 1A**). Activating receptor signaling converges on Syk/ZAP70 axis, orchestrating cytotoxic particle synthesis, trafficking, and exocytosis (9–11). Sustained activation further induces FasL/TRAIL expression, activating extrinsic apoptosis pathways in target cells (12). The immunomodulatory capacity of NK cells extends to cytokine production, with IFN-γ secretion enhancing macrophage activation and antigen presentation, while TNF-α release promotes both direct tumor apoptosis and inflammatory amplification (13). Cellular ontogeny studies reveal NK cell proliferation and differentiation depend on cytokine-regulated signaling cascades: The PI3K-Akt-mTOR axis governs developmental progression (14), while JAK/STAT activation sustains precursor cell expansion and survival (15) (**Figure 1B**). Intervention in the biological features of NK cells will be a great innovative help for treatment, which is crucial for patients with aggressive cancers.

**Figure 2:**
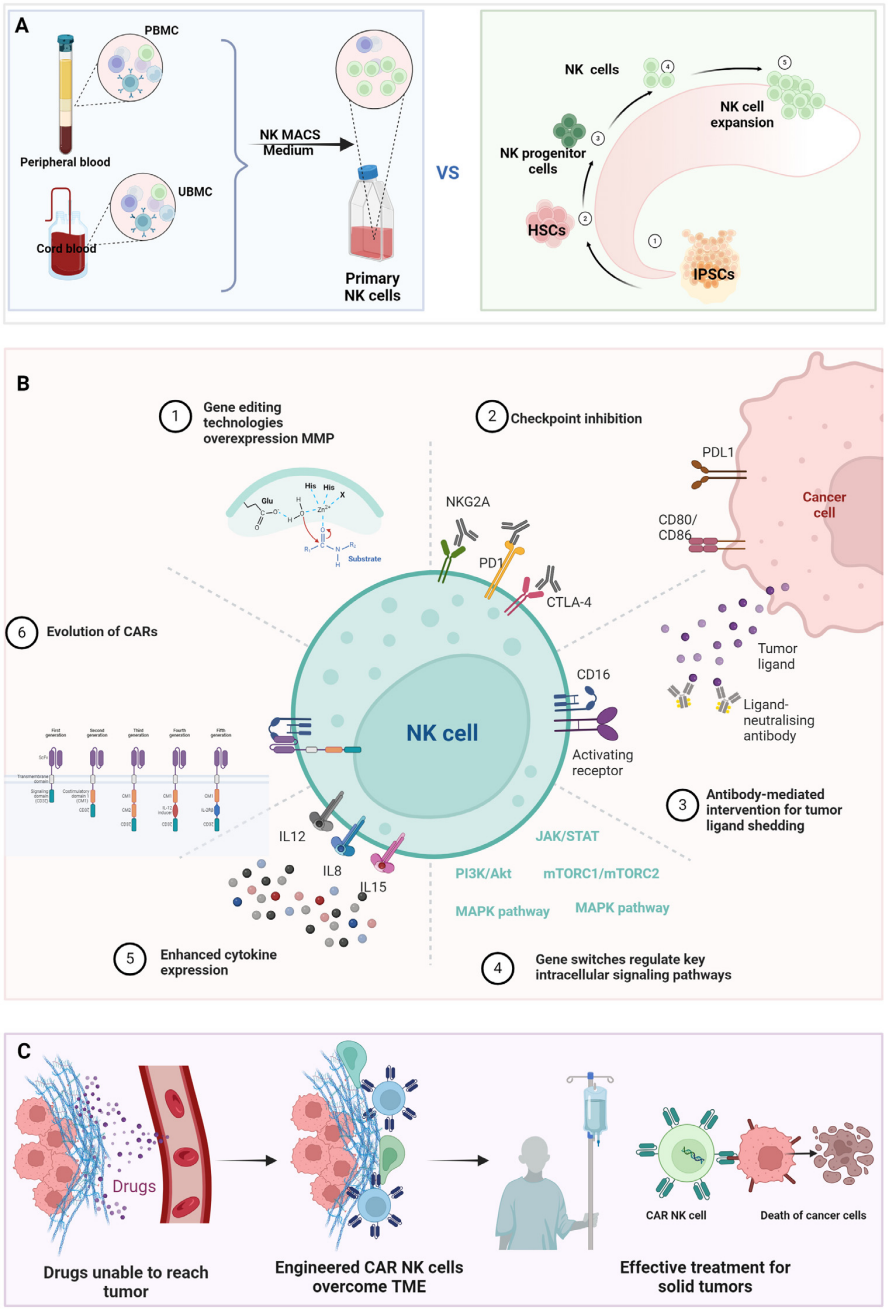
Bioengineering strategies to enhance NK cell antitumor efficacy. **(A)** Comparative profiles of NK cell sources: Functional and phenotypic differences among NK cell subsets derived from peripheral blood, umbilical cord blood, iPSC, or engineered cell lines. **(B)** Genetic engineering for functional optimization: (1). TME penetration: CRISPR-edited homing receptors (e.g., CXCR4/CXCR3) or matrix-degrading enzymes (MMPs); (2). Immune checkpoint modulation. (3). Knockout of inhibitory receptors (e.g., NKG2A) or overexpression of activating receptors (e.g., NKG2D, DNAM-1); (4). Intrinsic pathway activation: Constitutive signaling via STAT3, PI3K-AKT, or mTOR pathways; (5). Cytokine engineering: Armored CAR-NK cells secreting IL-15/IL-21 for autocrine survival; (6). CAR evolution: Multigenerational CAR designs (1st–5th gen). **(C)** CAR-NK-mediated TME reprogramming. (Figure created with BioRender).

Current NK cell sources exhibit distinct therapeutic tradeoffs (**Figure 2A**). NK cells originate from the bone marrow and can be derived from various sources, including peripheral blood, umbilical cord blood, NK cells lines, and induced pluripotent stem cells (iPSCs). Peripheral blood-derived NK cells demonstrate immediate cytotoxicity but limited persistence (16). Umbilical cord blood variants show enhanced expansion capacity through reduced cytotoxic potential (17). Immortalized cell lines offer superior scalability and effector function but suffer from poor *in vivo* survival and culture challenges (18). While iPSC-derived NK cell products provide theoretically unlimited expansion potential, they carry inherent tumorigenic risks and demonstrate genetic instability during prolonged *in vitro* culture processes (5). These critical safety concerns mandate comprehensive genomic stability monitoring, inducible safety genes, and rigorous tumorigenicity assessments through standardized assays (including teratoma formation tests and karyotype analysis) prior to clinical implementation (19). The inherent dichotomy between functional potency and practical manufacturability presents fundamental challenges in engineering optimal CAR-NK therapeutics. Emerging synthetic biology strategies aim to overcome these limitations through precision engineering of NK cell durability, functionality, and tumor-resistant phenotypes.

## Evolution of CAR architectures for NK cells

Chimeric Antigen Receptors (CARs) are synthetic constructs combining antibody-mediated specificity with cellular activation capacity, first successfully implemented in T cells. A canonical CAR architecture comprises three functional modules: an extracellular antigen-binding domain (scFv) for antigen recognition, a transmembrane domain, and an intracellular signaling domain [**Figure 2B** (6)]. First-generation CARs featured a solitary CD3ζ signaling domain that enabled antigen-specific activation but showed restricted clinical efficacy due to transient persistence (20). Second-generation designs incorporated a single co-stimulatory domain (CD28 or 4-1BB) alongside CD3ζ, markedly enhancing lymphocyte proliferation, metabolic fitness, and *in vivo* persistence – breakthroughs that revolutionized hematologic malignancy treatment (21, 22). However, these constructs remain vulnerable to immunosuppressive checkpoints and exhibit poor solid tumor penetration. Third-generation CARs addressed these limitations through dual co-stimulation (e.g., CD28 + 4-1BB), though excessive signaling occasionally precipitated T cell exhaustion via chronic activation (23, 24). Fourth-generation ‘armored’ CARs introduced inducible cytokine expression systems (e.g., IL-12) to remodel TME post-target engagement, though off-target cytokine release risks necessitated stringent safety controls (25). Fifth-generation platforms integrate tunable activation switches and fail-safe mechanisms, enabling real-time modulation of CAR activity to balance potency and toxicity (26, 27). This iterative engineering progression informs current CAR-NK cell development, which synergizes NK cells’ intrinsic advantages with synthetic biology precision editing.

## Synthetic biology toolkit witches regulate key physiological characteristics

The synthetic biology toolkits enable the systematic integration of genetic components that fine-tune NK cell behavior, amplify therapeutic efficacy, and impose spatiotemporal control over *in vivo* activity. A cornerstone of this approach involves rewiring intracellular signaling pathways governing antigen recognition, activation dynamics, and cytotoxic execution.

Critical to CAR-NK cell utility is target identification, where synthetic biology enhances ADCC through CD16 engineering. Genetic modifications enhancing IgG Fc binding affinity have demonstrated improved ADCC durability and potency (28). Currently, a CAR-NK cell product containing optimized CD16a is undergoing clinical evaluation. The preliminary results of the trial were encouraging: objective response rates of >50% in 17 patients, including 7 complete responses (29). Complementarily, synthetic receptor systems mimicking natural activating ligands like NKG2D counteract tumor immune evasion strategies driven by ligand shedding (30). Counter-regulation of inhibitory checkpoints further amplifies tumor targeting: CRISPR-mediated NKG2A ablation prevents HLA-E-mediated suppression, markedly enhancing cytotoxicity against resistant malignancies (31) [**Figure 2B** (2, 3)]. For precision targeting, synthetic biology is fundamentally transforming CAR-NK cell engineering by implementing logic-gated (OR, AND, and NOT) receptor architectures that enhance tumor specificity and mitigate off-target toxicity (32–34). OR-gated dual CAR systems employ independently functioning activation domains that synergistically activate NK cells upon recognition of either target antigen, effectively addressing tumor antigen heterogeneity (35). AND-gated CAR systems require sequential engagement of two distinct tumor antigens - primary CAR activation induces expression of a secondary CAR, creating a failsafe mechanism that spares healthy cells expressing single antigens. NOT-gated inhibitory CAR architectures incorporate immune checkpoint-derived signaling domains that dominantly suppress activation when encountering healthy tissue biomarkers (36).

Upon target engagement, synthetic circuits amplify cytotoxic payload delivery. Tet-On systems enable controlled perforin/granzyme release, synergizing with engineered soluble factor-related apoptosis-inducing ligand (TRAIL) expression to activate both intrinsic and extrinsic apoptosis pathways (37, 38). Cytokine engineering further augments antitumor immunity: chimeric receptors incorporating CD28/IL-12 signaling domains drive IFN-γ hypersecretion (39), while IL-21 co-expression potentiates cytotoxicity against epithelial tumors (40). In a clinical trial in which CD19 and IL-15 co-express CAR structures to improve survival rate, this therapy was effective and well tolerated in patients with lymphocyte clearance from a large number of relapsed or refractory CD19-positive malignancies, with a complete response rate of 64%. Besides, CAR-NK cells are detectable in the patient’s peripheral blood for more than 12 month (41).

Overcoming transient *in vivo* persistence remains paramount. PI3K/AKT pathway modulation via IL-15/CCL21 co-expression synergizes to sustain CAR-NK proliferative capacity and metabolic fitness (42, 43). In addition to increasing persistence, long term survival can also be achieved by introducing anti-apoptotic genes (such as BCL-2) to reduce activation-induced apoptosis (44). In terms of NK cells sources, iPSC-derived CAR-NK cells exhibit superior longevity compared to peripheral blood counterparts (45).

Safety engineering remains integral to clinical translation. Inducible caspase 9 (iCasp9) suicide switches enable rapid ablation during cytokine release syndromes (3). TME-responsive circuits, such as TGF-β-inducible CAR activation, prevent off-tumor toxicity by restricting effector function to malignant niches (46). Collectively, these synthetic biology innovations establish a robust framework for developing next-generation CAR-NK therapeutics with enhanced specificity, persistence, and safety profiles.

## Synthetic biology techniques reprogramming the TME

The TME suppresses immune cell cytotoxicity through three interconnected mechanisms (47–50). First, cancer-related fibroblasts secrete immunosuppressive factors including TGF-β, VEGF, IL-6, CXCL12, and PD-L1, while depositing extracellular matrix components like collagen and fibronectin that physically impede immune infiltration. Second, TME metabolites such as adenosine and lactate directly impair CD8+ T cell and NK cell effector functions. Third, aberrant tumor vasculature creates hypoxic niches that drive lactate accumulation via *Warburg* effect reprogramming, simultaneously fueling tumor proliferation and polarizing macrophages toward pro-tumoral M2 phenotypes through HIF-1α signaling. These immunosuppressive networks necessitate innovative strategies to enhance CAR-NK cell functionality within hostile TME (**Figure 2C**).

To enhance tumor infiltration, overexpression matrix metalloproteinase (MMP) engineering enables penetration through stromal barriers (51) [**Figure 2B** (1)]. Synthetic biology approaches counteract TME-driven immune evasion through multiple mechanisms. CRISPR-mediated knockout of inhibitory checkpoints like PD-1 on CAR-NK cells disrupts tumor immune resistance pathways (52) [**Figure 2B** (2,3)]. This modification can help overcome the immune evasion strategies employed by tumors, leading to improved therapeutic outcomes. TGF-β resistance has been achieved via CRISPR/Cas9-mediated TGFBR2 knockout, effectively neutralizing this potent immunosuppressive cytokine (46). Moreover, fused DNAX-activation protein 12 (DAP12) NK cells demonstrated improved efficacy and persistence in TGF-β–secreting TME (53).

Metabolic reprogramming strategies address TME-induced dysfunction: GLUT1 overexpression enhances glycolytic flux in hypoxic/lactate-rich conditions, sustaining CAR-NK cell metabolic fitness and survival (54). Advanced control systems incorporate hypoxia-responsive elements (HREs) to dynamically regulate CAR expression based on TME oxygen tension (55) [**Figure 2B** (4)]. Furthermore, engineered secretion of CCL5 enables CAR-NK cells to orchestrate antitumor immunity by recruiting T cells and dendritic cells [**Figure 2B** (5)], converting “cold” tumors into immunologically “hot” microenvironments, boots the cytotoxic of immune cells (34, 42). While these advancements demonstrate significant progress in overcoming TME constraints, clinical translation requires continued optimization of CAR-NK cell durability, specificity, and safety profiles through iterative synthetic biology innovations.

## Strategies combining other immunotherapies

The therapeutic potential of CAR-NK cells is amplified through strategic combination with complementary modalities that address tumor resistance mechanisms. Synergy with immune checkpoint inhibitors counteracts TME-driven immunosuppression, as evidenced by enhanced antitumor responses when combining PD-1/CTLA-4 blockade with CAR-NK cells through dual activation of innate and adaptive immunity (56). Combine cellular therapy approaches leverage distinct cytotoxic mechanisms: CAR-NK/CAR-T cell co-administration demonstrates improved solid tumor control via spatial cooperativity, where NK cells eliminate CAR-T-resistant antigen-low variants while T cells target bulk tumor populations (57). Traditional cytotoxic therapies potentiate CAR-NK efficacy through immunogenic modulation – radiotherapy-induced DNA damage upregulates NKG2D ligands while chemotherapy depletes immunosuppressive myeloid populations, collectively enhancing tumor visibility and NK cell infiltration (58). This multidimensional strategy surmounts tumor heterogeneity by simultaneously targeting malignant clones, stromal barriers, and immune evasion pathways.

## Future research

The clinical application prospects for CAR-NK cells are robust, with ongoing research expected to yield innovative approaches that enhance their therapeutic potential in cancer treatment. Innovation in synthetic biology enables researchers to design and engineer NK cells with unprecedented specificity and functionality. The field of synthetic biology is poised to well benefit the development and application of CAR-NK cells in cancer therapy.

Synthetic biology can facilitate the development of modular CAR constructs that can be easily adapted to target various antigens, thus streamlining the process of CAR design and production (30). Traditional CAR therapies typically focus on a single target antigen, which can lead to tumor escape mechanisms, especially in heterogeneous cancers where antigen loss is common. Dual-target CAR-NK cells can simultaneously engage multiple tumor-associated antigens, thereby reducing the likelihood of tumor evasion and enhancing overall therapeutic efficacy (32). The ability to design CARs that can recognize multiple antigens simultaneously is particularly valuable in treating complex malignancies, such as acute myeloid leukemia, where antigenic heterogeneity poses a significant challenge. In addition, adding a protective CAR to prevent normal cells from being accidentally injured is also a suitable way. Additionally, the integration of artificial intelligence (AI) with synthetic biology is fundamentally transforming CAR-NK cell development through many key mechanisms (59, 60): optimizing structures with algorithms; customizing personalized treatment regimens; and imitating treatment effects. Through the integration of AI technologies, CAR-NK design will shift from a “trial-and-error mode” to a “prediction-verification-optimization” closed loop, significantly accelerating the development of next-generation therapies. However, there is no ‘off of shelf’ NK cell in the market. Critical challenges persist in manufacturing scale-up and safety assurance. Good manufacturing practice-compliant scale-up requires standardized protocols for closed-system bioreactor platforms and automated cell processing systems. Safety assurance demands the implementation of multiplexed pathogen and residual pluripotent cells testing (61, 62). While iPSC-derived CAR-NK platforms progress toward off-the-shelf availability, standardized potency metrics, and reduced *ex vivo* manipulation costs remain prerequisites for global accessibility. Long-term monitoring must address theoretical risks of insertional oncogenesis from viral vectors and HLA-independent alloimmunity. The field requires harmonized clinical-grade production protocols validated through multicenter trials, coupled with real-world evidence frameworks tracking durable remission patterns.

Beyond CAR-NK cell optimization, synthetic biology has revolutionized microbial-based cancer therapeutics through two groundbreaking approaches: tumor-targeted bacterial vectors (63) and xenogeneic antigen engineering (64), these have greatly broadened the path of tumor therapies.

Through convergent innovation across synthetic biology, and computational immunology, optimized NK sources, CAR-NK cells are poised to transcend niche applications from robust hematologic malignancy control to engineered tissue-homing systems addressing solid TME. Realizing these potential demands sustained interdisciplinary collaboration to navigate the intricate interplay between cellular physiological characteristics, tumor adaptability, and host immunity.
